# Time for a Change: Why Psycholinguistics Should Move Beyond the Content-Function Word Distinction

**DOI:** 10.1007/s10936-026-10306-0

**Published:** 2026-09-11

**Authors:** Solvej Wilbrandt Jaeger, Kasper Boye

**Affiliations:** Department of Nordic Studies and Linguistics, University of Copenhagen, Njalsgade 136 & Emil Holms Kanal 2, Copenhagen S, 2300 Denmark

**Keywords:** Eye-tracking, The lexical-grammatical contrast, Reading, Attention

## Abstract

The traditional distinction between content and function words continues to underpin much psycholinguistic research, even though other areas of linguistics have long recognized it as problematic (e.g. Bennis et al., 1983, and Ishkhanyan et al., 2017 within aphasiology; Diessel, 1999: 150–153, and Bisang, 2010: 291 within linguistic typology; Emonds, 1985: 162–164, and Rauh, 1993 within grammatical theory). We argue that relying on this pre-theoretical distinction in psycholinguistics can obscure meaningful variation and potentially lead to misleading results. We illustrate this with Staub (2024), who, in a large-scale eye-tracking study, found no evidence that so-called function words are skipped more often or receive shorter fixations than so-called content words. We argue that this null finding likely stems from Staub’s function word group being heterogeneous, containing both lexical and grammatical items. In support of this, we reclassify the words in Staub’s function word group as either lexical or grammatical based on a usage-based theory of the lexical-grammatical contrast (Boye & Harder, 2012), and rerun the analysis in his Experiment 1 on this group of words. Our analysis suggests that the grammatical words receive shorter fixations than the lexical words, providing support for the heterogeneity of the function word group. Given this, it is unsurprising that Staub found no differences in reading measures between the function and content word categories. This case demonstrates how relying on the traditional function-content distinction in psycholinguistic research can obscure meaningful patterns, and it highlights the potential of adopting Boye and Harder’s (2012) usage-based alternative.

## Introduction

It is a widespread and longstanding assumption that words can generally be divided into two categories: content words and function words. This distinction is deeply embedded in the field of psycholinguistics, where it often underpins experimental designs and data interpretation. However, in this paper we will argue that the traditional content-function word distinction is inherently problematic and that basing psycholinguistic research on it introduces a fundamental source of error.

A recent study by Staub (2024) provides a clear example of the problems that can arise from relying on this traditional content-function word distinction. In two large-scale eye-tracking experiments, the difference in eye movement behavior on content and function words was assessed. Stimulus sentences were selected from a corpus of natural text, and length, frequency, and predictability of the target words were carefully controlled. In Experiment 1, content and function word targets with low predictability were contrasted. In Experiment 2, also contexts that rendered the target words fairly predictable were included. Neither experiment yielded evidence that function words were skipped more or received shorter fixations than content words.

However, we will argue that Staub’s (2024) results are most likely influenced by the fact that the group of words classified by him as function words is in fact heterogeneous, containing both content and function words, or rather what we will call lexical and grammatical words (see below). In this paper, we use Staub’s (2024) study as a case in point to show how relying on the traditional content-function distinction can obscure meaningful variation in psycholinguistic data.

Our main aim is purely conceptual: we point out problematic aspects of the traditional content-function word distinction and present the usage-based distinction in Boye and Harder (2012) between lexical and grammatical items as an alternative. This alternative distinction is intended to capture the same basic intuitions as the traditional content-function word distinction, namely that there is a semantic difference between on the one hand, lexical (or content) items like nouns and verbs, and on the other hand, grammatical (or functional items) like articles, auxiliaries, affixes and syntactic constructions. However, the lexical-grammatical distinction in Boye and Harder (2012) does not share the problems of the traditional content-function distinction, and it draws the dividing line between the meaning of lexical and grammatical items slightly, but significantly, differently.

In support of our main aim, we subsequently reclassify Staub’s function words into a lexical and a grammatical subgroup, and rerun the analysis in Staub’s Experiment 1 on those subgroups, showing that there is indeed a difference between them. We need to emphasize that due to the fact that our analysis only includes a subset of Staub’s words, the analysis, while showing a significant difference between lexical and grammatical words, can and should only be seen as a supportive supplement to our main, conceptual argument.

We round off the paper with a discussion of our results, and in our conclusion, we emphasize the need for psycholinguistics to move beyond the traditional content-function word distinction, and base studies of the lexical-grammatical contrast on a coherent and empirically supported theory.

## The Traditional Distinction Between Content and Function Words and its Problems

Table 1 lists the content and function words selected by Staub (2024).

**Table 1:** Content and function words in Staub (2024)

Content	Function
Come	About
Girl	Before
Good	From
Know	Here
Little	Never
Love	Some
Make	Then
Money	These
Name	They
Night	When
People	Where
Think	Will
Time	With
Want	Would

Staub reports that he “attempted to choose words that fall unambiguously into the function or content class based on common criteria” (Staub, 2024, p. 971). Specifically, he here refers to the criteria used by Bell et al. (2009). In what follows, we point out a number of unsurmountable problems in this approach, problems that have long been demonstrated (see Boye 2023a for a recent discussion).

Two basic problems concern the distinction between content and function words that Staub’s approach relies on. Firstly, the distinction embodies an assumption that there are two ontologically different kinds of word semantics: (1) conceptual or referential “contents” that are directly activated by the words that have them, and (2) procedural “functions” that operate on those contents. This assumption is not only pretheoretical, but also rejected by leading scholars in both generative linguistics (Friederici et al., 2017) and functional-cognitive linguistics (Harder, 1996; Evans 2009). These scholars argue that also the meanings of content words like *cat*,* swim* and *yellow* are in fact procedural or functional in the sense that they are instructions for mental operations (Friederici et al., 2017: 716; Harder, 1996:111–115 for a more thorough discussion). Secondly, for all practical-analytical purposes, the notions of content and function are too vague to be of any use, and need to be translated into something more helpful. Definitions of the notions therefore standardly refer to open and closed classes respectively (e.g., Martinet, 1960: Sect.  4.19; Segalowitz & Lane, 2000; Harley, 2006: 118). This is the case also with the definition in Bell et al. (2009: 92) which is adopted in Staub (2024: 971).

This second distinction between open and closed classes is empirically clear: open classes are those that readily accept new members, closed classes are those that do not. Thus, in English, the word classes of nouns (e.g. *cat*, *sweater*) and adjectives (e.g. *little*, *blue*) are examples of open classes, while the word classes of articles (e.g. *the*, *a(n)*) and prepositions (e.g. *off*,* of*) are examples of closed classes. But it is far from clear that this distinction is of any theoretical interest. For one thing, what belongs to open and what to closed classes is a language-specific matter. While European languages have open classes of adjectives, other languages have closed classes (Schachter & Shopen, 2007). In many modern European languages, likewise, person names form open classes, but classical Latin praenomina (defined syntactically by taking the first of three positions in the person name construction) constituted a closed class.

Another argument that the open-closed class distinction is not of much theoretical interest is that both open and closed classes may be rather heterogeneous in terms of the syntactic and semantic properties of their members. On the one hand, open classes may comprise members that would intuitively be classified as function words. In English, for instance, verbs comprise auxiliaries (e.g. *have* as in *I have lost my cat*, and *do* as in *Did you lose your cat?*) as a subcategory, and already Emonds (1985: 162–164) argued that functional “subclasses” can be identified for nouns, verbs, adjectives and prepositions. On the other hand, closed classes may comprise members that, due to their syntactic and semantic properties, must be categorized as content words. Accordingly, there is a rather long tradition for analyzing English prepositions as including not only function words (e.g. *of*, *for*), but also content words (e.g. *off*,* before*) (e.g. Rauh, 1993; Littlefield, 2005).

What is perhaps most important in the present context is that the psycho- and neurolinguistic evidence for an independent effect of open- or closed-class membership is mixed and does not provide a consistent predictive pattern. This is clear above all from a series of studies in aphasiology that began already in the early 1980’s, and which shows that different subclasses of prepositions behave differently in aphasia. For instance, Bennis et al. (1983) showed that the heterogeneity of adpositions is reflected in aphasic speech: individuals with Broca’s aphasia underproduced Dutch prepositions classified as “syntactic” relative to prepositions classified as “lexical” or “subcategorized”, whereas individuals with Wernicke’s aphasia showed a tendency in the opposite direction (see also Friederici, 1982 and Bastiaanse & Bennis, 2018). More recently, Ishkhanyan et al. (2017) found that the production of French pronouns classified as grammatical was more reduced in Broca’s aphasia than pronouns classified as lexical when compared to the production in neurotypical speech. Similar results have been found by Boye & Bastiaanse (2018) for lexical vs. grammatical verbs in Dutch, and by Nielsen et al. (2019) for lexical vs. grammatical determiners in Danish (that is, indefinite articles vs. determinative numerals corresponding to English *a(n)* vs., *one*). A parallel outside aphasiology is Foucambert and Zuniga’s (2012) letter detection experiment which found that more letters were detected in prepositions categorized as “full” than in prepositions categorized as “empty”, suggesting that the former attract more attention (see further discussion below).

Taken together, these findings suggest that the open- vs. closed-class distinction does not in itself provide a consistent predictive pattern, as relevant differences may instead emerge between subclasses within these categories. Staub’s (2024) study fits into this broader picture, as it does not show robust or consistent associations between the open- vs. closed-class distinction (or the distinction between content and function words based on the former distinction) and behavioral eye-tracking measures.

Our claim is that this does not exclude that a more well-understood and theoretically anchored distinction between content and function items – or as we shall prefer, lexical and grammatical elements – does have predictive power. In fact, the studies mentioned above that found differences between lexical and grammatical verbs, pronouns and determiners are based on a theoretically solid distinction between lexical and grammatical elements. Accordingly, we would hypothesize that to the extent that the numerous existing psycho- and neurolinguistic studies of the open- vs. closed-class distinction found any differences between the two classes, it is due to the fact that the groups of words contrasted happen to show a considerable overlap with the more solid distinction between lexical and grammatical elements. In the next section, we will outline this more solid distinction.

## A Usage-Based Theory of the Lexical-Grammatical Contrast

According to the usage-based theory outlined in e.g. Boye and Harder (2012); Boye (2023b), 2024), the lexical-grammatical contrast is a means for coordinating attention between speakers. Thus, it may be conceived of as related to means for foregrounding parts of linguistic messages (e.g. emphatic stress, clefting, focus particles) as well as to means for backgrounding such as parentheses in the written language. What is special about the lexical-grammatical contrast is that it is built into the building blocks of utterances (meanings, morphemes, words, phrases) rather than superimposed upon them. Also, the contrast is asymmetric. Lexical elements (whether meanings, morphemes, words or phrases) are conventionalized, hence entrenched, with the potential to be the attentional foreground of a larger whole (ultimately an utterance), but whether they are so or not depends on the context. In contrast, grammatical elements are conventionalized as background information. Consider, for instance, the utterance,

*Stop smoking!*

This utterance consists of two lexical words, and either of these can be foreground. In a context where attitudes towards smoking are discussed, *stop* would express the foreground information, whereas *smoking* would likely be foreground if the topic were ‘things you should stop doing’. Consider now the grammatical affix *-ing* in the same utterance. This affix will always be background. The attentional main point of saying *Stop smoking!* will never be to convey that an action is construed as ‘progressive’ (or whatever the meaning of -*ing* is). Rather *-ing*, as a conventionalized background element, cannot stand alone (*Ing!), but depends on combination with a host relative to which it is backgrounded. *-Ing* and other grammatical elements can only be foregrounded when conventions are overridden as in metalinguistic contexts like *I said jumpED*.

In short, lexical and grammatical elements are defined as follows,

- Lexical elements are by convention foregroundable.
- Grammatical elements are by convention backgrounded.

### Psycho- and Neurolinguistic Support

The above definitions constitute the backbone of a theory of grammaticalization as a special kind of conventionalization (Boye 2023a, 2024) and a theory of grammatical impairment as a compensatory response to cognitive resource reduction (Boye et al., 2023). Also, the definitions entail hypotheses about language production and language perception that are supported by a range of recent studies of both language perception and language production, and of both neurotypical and grammatically impaired speech.

As for language perception, the definitions entail that grammatical elements attract less attention than lexical ones, also when frequency is controlled for.^1^ Among several studies that found support for this hypothesis, Christensen et al. (2021) conducted a text-change experiment and found that participants detect fewer word omissions when the word being omitted is grammatical (and attracts less attention) than when it is lexical (and attracts more). Also, a recent eye-tracking experiment found support for the hypothesis in a reading study of Kalaallisut (Greenlandic) affixes (Jaeger et al., sbm. ; see Discussion).

As for language production, the definitions entail that production of grammatical words is associated with longer reaction times, and – if working memory is loaded – more errors. Michel Lange et al. (2017) found support for the first hypothesis in a study which contrasted the production of Danish counterparts of the full verb *have* as in *I have a stolen car* and auxiliary *have* as in *I have stolen a car*. As for the hypothesis concerning error, Ishkhanyan et al. (2019) found support for this in a study which contrasted the production of lexical and grammatical words in persons who were simultaneously performing a span task. Danish lexical numerals and grammatical articles were contrasted in noun phrases corresponding to English *one/an orange letter*, and the grammatical articles were associated with far more deviations (mostly omissions) than the lexical ones.

Finally, classifications of words into lexical and grammatical ones based on these definitions have been shown to be significant for the description of grammatically impaired speech across word classes and several languages (e.g. Ishkhanyan et al., 2017, on French pronouns; Boye & Bastiaanse, 2018, on Dutch verbs; Nielsen et al., 2019, on Danish determiners; Bau & Boye, 2023, on English prepositions). In all these studies, words classified as grammatical are underproduced in grammatically impaired speech, while this is not the case for words classified as lexical. In a study parallel to the one by Ishkhanyan et al. (2019) mentioned above, for instance, Nielsen et al. (2019) contrasted the production of lexical numerals and grammatical articles in persons with non-fluent aphasia and found that the grammatical articles were omitted far more frequently than the lexical ones.

### Criteria

Classifications of words into lexical and grammatical ones are directly based on a set of criteria entailed by the definitions given above. Notably, the definition of lexical elements as foregroundable entails that they can be focused and modified. In contrast, it follows from the definition of grammatical elements as backgrounded by convention that they cannot be focused or modified. Neither of these criteria are new. It has long been recognized that grammatical elements resist focusing and modification (e.g. Bybee et al., 1994; Keizer, 2007). What is new is only that the theory outlined here brings the two features into a motivated relationship. The criteria while language-general are subject to language and word-class specific restrictions and depend on language and word-class specific means for focusing and modifying. In the case of English, for instance, most means for focusing – clefting, pseudo-clefting, focus particles – do not work below constituent level.

As our reclassification of Staub’s function words depends on these criteria, we will introduce them in a little more detail. In the sense relevant here, modification is a semantic operation in which one meaning adds to another meaning in its scope. (1) may serve as an example.

1. Sue has secretly stolen a car.

In (1), the meaning of *secretly* takes the meaning of *stolen* in its scope and adds to it, specifying the manner in which Sue stole something. This shows that *stolen* can be modified, and thus, that it is a lexical word. In contrast, the perfect auxiliary *has* is not modified by *secretly*. The meaning of the perfect auxiliary can be described as ‘anterior with present relevance’, and this meaning cannot be modified by the meaning of a manner adverb nor by the meaning of any other word or morpheme. This shows that the auxiliary is grammatical.

Of course, the perfect meaning may occur in the scope of a sentence adverb like *definitely* or *probably*, but as the name ‘sentence adverb’ suggests, these adverbs do not specifically target the perfect meaning of *has*. That is, they do *not only* take the perfect meaning in their scope, and they do *not only* add semantically to that meaning; rather, they modify the entire proposition expressed by the sentence.

As for the focus criterion, it is illustrated in (2).

- It is that that I like.

(2b) *It is it that I like.

(2a) shows that the English pronoun *that* can be focused by means of clefting, which is evidence that this pronoun is lexical. However, as shown in (2b), the pronoun *it* cannot be focused by means of clefting, which suggests that *it* is grammatical.

As in the case of the modification criterion, the focus criterion is basically semantic. That is, it basically applies to meaning. Thus in (2), *that* is focused in the sense that its meaning (that is, its demonstrative reference) is singled out and foregrounded. In contrast, stressing an auxiliary, as in *Sue HAS stolen a car* does not focus the auxiliary semantically. Rather, such stressing – known as *verum focus* – focuses the whole proposition, emphasizing its truth.

In what follows we use these criteria to reclassify Staub’s target words. It must be stressed that meeting one criterion suffices to be classified as lexical. Classification as grammatical requires both non-focusability and non-modifiability.

## Reclassification of Words

Staub’s target words can be reclassified as either lexical or grammatical based on the above criteria. For example, the meaning variant of the preposition *before* in (3) is modifiable by means of *right* and must therefore be lexical (cf. e.g. Rauh, 1993, mentioned earlier)^2^:

(3) You don’t have to have it all figured out (*right*) before you get started.

Conversely, the meaning variant of the preposition *with* is not modifiable, as illustrated in (4). Since moreover, it cannot be focused, it must be classified as grammatical:

(4) If you could be penpals (*right) with anyone, living or dead, who would you write to?

The focus and modification criteria support Staub’s classification of content words. All these words are either modifiable or focusable or both modifiable and focusable. What is important here, however, is that more than half of Staub’s function words are actually lexical according to these criteria. This is shown in Table 2, which gives the results of our reclassification of Staub’s function words.

**Table 2:** Recategorization of Staub’s (2024) function word group

Grammatical	Lexical	Both a lexical and a grammatical variant
With	Here	Some
Will	Never	From
Would	Before	
About	Where	
	When	
	These	
	Then	
	They	

A few of the target words (*some* and *from*) have both a lexical and a grammatical meaning variant. In these cases, we categorized the occurrences of the words in Staub’s stimulus sentences separately. For example, *from* as an expression of ‘location’ in (5) is modifiable and therefore lexical:

(5) They both end up crashing through the table (*right*) from the top of the cage.

In contrast, *from* as an expression of ‘source’ in (6) is arguably non-modifiable, and since it arguably cannot be focused, it must be considered grammatical (Bau & Boye, 2023):

(6) That’s because 90% of the heat energy (*right) from climate change goes into the oceans, Willis said.

A few of the stimulus sentences had to be excluded from analysis, as the target word could not be unambiguously classified as lexical or grammatical. In these cases, the basic problem is that the target word in hand has both a lexical and a grammatical meaning variant, and that the context does not disambiguate the word. That was for example the case with *some* in (7):

(7) We have ((*)*only*) some confident freshmen that have bought in, but they are a humble group too.

If *some* here is understood as designating an indefinite quantity (what Cambridge Dictionary refers to as ‘the weak form of some’^3^), it is unmodifiable and thus grammatical. However, in this sentence, *some* can also be understood as contrasting with *all* or *enough* (what Cambridge Dictionary calls ‘the strong form of some’). In the latter case, it is modifiable by means of *only* and thus lexical. It is not possible to determine whether a given participant interpreted *some* here as one or the other. Therefore, we excluded the sentence completely from our analysis.

## Hypotheses

We have argued that Staub’s function word group is heterogeneous, containing both lexical and grammatical words. Based on the theory outlined in Boye and Harder (2012); Boye (2023a, 2024), we would expect to see a difference in eye movement behavior between these two groups.

As mentioned above, the theory entails that grammatical elements attract less attention than lexical ones in language perception. This has been confirmed in various psycholinguistic studies (e.g. Christensen et al., 2021; Klein & Saint-Aubin, 2016). For eye-tracking, this leads to the prediction that grammatical elements will receive less visual attention (i.e. fewer fixations and shorter fixation times) than lexical elements in reading.

We therefore hypothesize that:

1. Grammatical words are skipped more frequently than lexical words.

(2) Grammatical words receive shorter fixations than lexical words.

In the next section, we will go through our reanalysis of Staub’s (2024) data.

## The Reanalysis: What We Did

In order to test the above hypotheses, we reanalyzed a portion of the data from Staub’s (2024) Experiment 1. Our goal was to examine whether the group of words categorized by Staub as function words was in fact heterogeneous, containing both lexical and grammatical words. More specifically, we asked whether reclassifying the words as lexical and grammatical would reveal meaningful differences in skipping and fixation time measures that were not apparent in Staub’s original analysis. We focused on Experiment 1 rather than both experiments because the present work is not intended as a full replication of Staub (2024), but as a targeted reanalysis used to illustrate the theoretical argument, and because Staub reports broadly similar patterns across experiments.

Staub focused on three eye movement measures in his analysis: skipping probability on the first pass, first fixation duration, and gaze duration (the sum of all eye fixation durations on the word on the first pass). Together, these measures capture first-pass processing of the target word. To analyze the data, Staub constructed logistic or linear Bayesian mixed-effects models using the brms package in R (Bürkner, 2017). The reading time measures were log-transformed. Each model was made available in full in the analysis scripts in Staub’s data repository. This allowed us to use exactly the same models for our analysis.

Since, as mentioned, the aim of the reanalysis was to examine the potential heterogeneity of the function-word group, we restricted our analyses to this set of words. We could in principle have included all items in the reanalysis, as Staub’s content words can be classified as lexical under the present framework. However, including the full dataset was not necessary to address the research question and would introduce additional noise and variation due to systematic differences in word-class distributions between the function and content groups. By focusing on only the function-word set, we could directly test whether this category was internally heterogeneous with respect to the lexical-grammatical distinction without conflating it with broader word-class differences. In addition, a subset of the content words in Staub’s materials show particularly short fixation durations and appear to contribute disproportionately to the observed patterns. Focusing on the function-word set therefore also reduces sensitivity to these effects. We return to this issue in the Discussion.

Table 3 reports descriptive statistics (means and standard deviations) for all variables of interest after reclassifying Staub’s (2024) original function word set into lexical and grammatical words.

**Table 3:** Mean item statistics (SD) of stimulus sentences by target word class after the new grouping

Measure	Grammatical	Lexical
Entropy	4.14 (1.09)	4.08 (1.24)
Number of preceding words	7.69 (3.69)	7.53 (3.31)
Preceding word length	5.10 (1.91)	4.83 (2.24)
Log target predictability	-1.65 (0.58)	-2.06 (0.54)
Target length	4.41 (0.49)	4.58 (0.69)
Preceding skip	0.34 (0.47)	0.39 (0.49)
Target zipf frequency	6.43 (0.18)	6.26 (0.25)

**Fig. 1:**
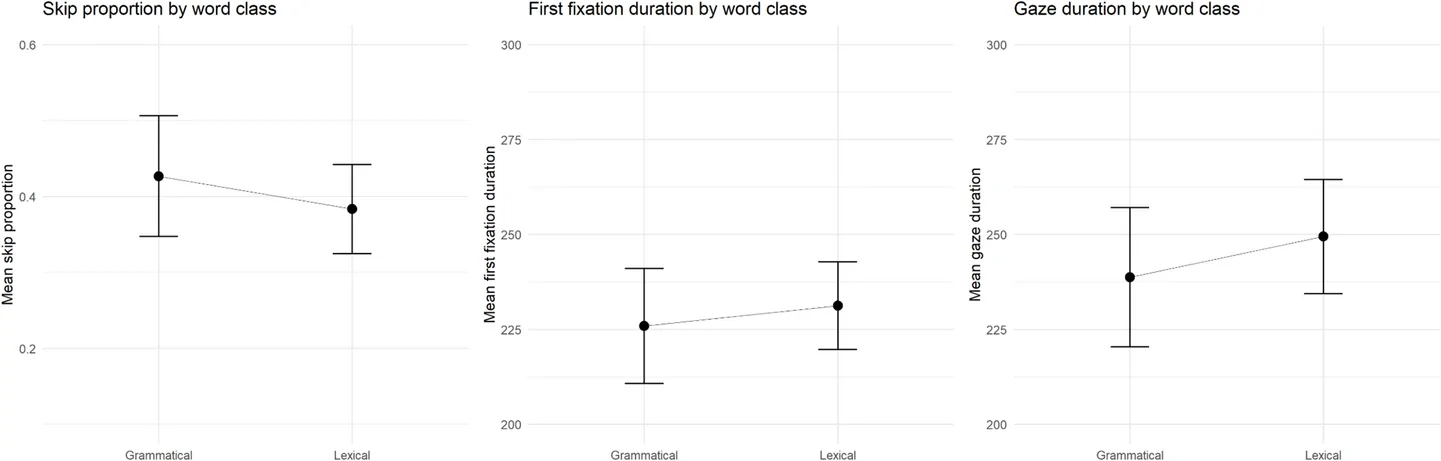
Skipping proportion, first fixation duration, and gaze duration means for the recategorized grammatical and lexical words in Staub’s (2024) function word group, with by-subject standard errors

Figure 1 shows skipping proportion, first fixation duration and gaze duration means for the recategorized lexical and grammatical words in Staub’s function word group. All show numerical trends in the expected direction, with more skipping of grammatical words than lexical words and shorter fixations on grammatical words than lexical words.

We based our analysis of each of the three measures closely on the linear Bayesian mixed-effects models presented by Staub (2024). The results of these will be presented in the following sections.

## Results

The reanalysis script is available at: https://github.com/solvej-jaeger/Reanalysis_Staub_2024.

### Word Skipping

Our initial model included eight fixed effects. Seven of these were the same as in Staub’s (2024) analysis: word class (sum coded with lexical word as -0.5 and grammatical word as 0.5), entropy, target word position, preceding word length, log target word predictability, target word length (all centered), and preceding word skip (binary predictor with fixation on preceding word coded as -0.5 and skip coded as 0.5). We also included centered Zipf frequency (which was closely matched in Staub’s design, but not in ours after our reclassification of the target words). We excluded function word probability as a predictor, because this measure is based on Staub’s definition of function words which differs from our definition of grammatical words. There was therefore no theoretical basis for including the predictor in our analysis.

Similarly to Staub (2024), for the effect of word class, we specified a Gaussian prior with a mean of 0 and a SD of 0.3 logit units. The model included random intercepts for subjects and items, and random by-subject slopes for the effect of word class (also similarly to Staub’s model).

The initial model revealed no hint of an effect of entropy or log target predictability. By this we mean that the posterior estimates were close to 0 with credible intervals spanning 0 and no systematic directional pattern. As these two predictors were not central to the hypothesis and did not contribute meaningfully to explaining variation in skipping, they were excluded, and we computed a second model with the remaining six predictors. The results of that model are shown in Table 4.

**Table 4:** Estimates from Bayesian mixed-effects logistic regression model of skipping probability

Effect	Estimate	Estimate error	Lower 95%	Upper 95%
Intercept	**-0.85**	**0.11**	**-1.06**	**-0.63**
Word class	0.08	0.10	-0.11	0.27
Number of preceding words	**0.09**	**0.01**	**0.06**	**0.12**
Preceding word length	**-0.09**	**0.02**	**-0.13**	**-0.04**
Target word length	**-0.75**	**0.10**	**-0.94**	**-0.56**
Preceding word skip	**-2.30**	**0.07**	**-2.43**	**-2.16**
Target Zipf frequency	-0.38	0.26	-0.90	0.14

Parameter estimates with credible interval that does not include 0 are in bold

The mean of the posterior distribution for the effect of word class is in the direction of more skipping of grammatical words, but the credible interval on this estimate extends on both sides of 0. There is thus no strong evidence for an effect of word class on skipping.

### First Fixation Duration

Our initial model again included all eight predictors mentioned above, and had the same random effect structure. Again similarly to Staub (2024), we assumed a Gaussian prior on the effect of word class with a mean of 0 and a standard deviation of 0.05 log units, which corresponds to approximately 10 ms. The initial model showed no meaningful contribution of entropy, number of preceding words, preceding word length or log target predictability to explaining variation in first fixation durations, with posterior estimates close to 0 and credible intervals spanning 0. These predictors were therefore excluded for parsimony.

We constructed a second model with the remaining four predictors. The fixed effect estimates are shown in Table 5.

**Table 5:** Estimates from Bayesian mixed-effects logistic regression model of first fixation duration

Effect	Estimate	Estimate error	Lower 95%	Upper 95%
Intercept	**5.38**	**0.01**	**5.35**	**5.40**
Word class	**-0.03**	**0.01**	**-0.05**	**-0.00**
Target word length	0.02	0.01	-0.00	0.04
Preceding word skip	**0.03**	**0.01**	**0.02**	**0.05**
Target Zipf frequency	0.03	0.03	-0.04	0.10

Parameter estimates with credible interval that does not include 0 are in bold

The mean of the posterior distribution for the effect of word class is in the direction of longer first fixations on lexical words, as predicted by our hypothesis, and the credible interval just excludes zero.

### Gaze Duration

As Staub (2024) points out, the target words were quite short and frequent and therefore often processed in a single fixation. It is therefore to be expected that the gaze duration results are somewhat similar to the first fixation duration results.

We again constructed an initial model with all eight predictors, using the same prior for the effect of word class as we did for first fixation duration. Three predictors showed no hint of an effect, with coefficients close to 0 and credible intervals spanning 0, and were thus excluded for parsimony: entropy, number of preceding words, and length of preceding word. The results from the second model with the remaining predictors are shown in Table 6.

**Table 6:** Estimates from Bayesian mixed-effects logistic regression model of gaze duration

Effect	Estimate	Estimate error	Lower 95%	Upper 95%
Intercept	**5.42**	**0.02**	**5.39**	**5.46**
Word class	**-0.04**	**0.02**	**-0.07**	**-0.01**
Target word length	**0.04**	**0.01**	**0.01**	**0.06**
Log target predictability	-0.02	0.01	-0.04	0.01
Preceding word skip	**0.03**	**0.01**	**0.01**	**0.06**
Target Zipf frequency	0.06	0.04	-0.02	0.13

Parameter estimates with credible interval that does not include 0 are in bold

Just as in the analysis of first fixation duration, the mean of the posterior distribution for the effect of word class is in the direction of longer gaze durations for lexical words, as predicted by our hypothesis, and the credible interval excludes zero.

Importantly, because these reanalyses rely on a relatively small set of target words, we also conducted leave-one-word-out robustness analyses for the first fixation duration and gaze duration models. The models were refit repeatedly while excluding each target word in turn. The estimated effect of word class remained negative in all analyses and was similar in magnitude across exclusions (estimates ranged from − 0.02 to -0.04 for the first fixation duration model and from − 0.02 to -0.05 for the gaze duration model). In most cases, the 95% credible interval continued to exclude zero. This suggests that the observed effect was not driven by any single lexical or grammatical word.

## Discussion

Staub (2024) found no evidence that function words are skipped more often or receive shorter fixations than content words when frequency, length, and predictability are controlled for. However, our reanalysis of data from the function word group in Staub’s Experiment 1 suggests that the words classified as lexical under the criteria presented here do receive longer fixations than those classified as grammatical. This finding supports our claim that Staub’s function word group is heterogeneous, containing both lexical and grammatical words. With this in mind, it is unsurprising that Staub (2024) did not detect differences in reading measures between the function and content word groups.

A similar issue is raised in other studies, e.g. in Foucambert and Zuniga’s (2012) letter-detection study of French. They found that prepositions, as a group, exhibited a rate of missed letters that fell between function words (such as determiners, conjunctions, and relative pronouns) and content words (such as nouns, adjectives, and adverbs). However, when prepositions were divided by “semantic load,” “empty” prepositions behaved like function words, while “full” prepositions behaved like content words. Despite this, Foucambert and Zuniga (2012:41) maintained that “[p]repositions are indeed function words,” albeit “a class of their own.” We would argue instead (in line with e.g. Rauh, 1993; Littlefield, 2005; see above) that their preposition group is heterogeneous, containing both lexical and grammatical members – just as Staub’s function word group. This illustrates how failing to recognize the lexical-grammatical distinction within word classes can obscure meaningful differences in reading measures.

Another relevant study to mention is a recent investigation of content and function word reading in Brazilian Portuguese (Vieira et al., 2024). Like Staub (2024), the authors report only minimal differences in fixation times between the two word groups, although they do observe differences in skipping rates for the short words. These mixed results could potentially, as in Staub (2024), be influenced by the way the content and function words have been classified.

It is however less straightforward to test this for Vieira et al.’s study than for Staub’s. The reason is that while Staub based his study on rather carefully chosen sentences including his target words, Vieira et al.’s study is based on a corpus of natural reading. This means that the category of word forms that are ambiguous and have both a lexical and grammatical variant needs to be determined token by token. Consider the word form *have* as an example. When it occurs as a full verb in construction with an object, as in *I have a book*, it must be classified as a lexical word (in our sense) or a content word (in the traditional sense; cf. the fact that together with other transitive verbs it belongs to the open class of verbs). But when it occurs as an auxiliary in construction with a participle, as in *I have arrived*, it must be classified as a grammatical word or function word.

Such classifications obviously become more time demanding if they are based on a fine-grained distinction between lexical and grammatical that allows for more cases of ambiguous words. But there is no easy way out: Ambiguous forms are abundant and a natural consequence of the grammaticalization processes through which lexical sources give rise to new grammatical descendants (e.g. Boye 2023b, 2024). As illustrated by the development of auxiliary *have* from the homonymous full verb, sources and descendants co-exist for some time, and in this period of time there will be ambiguity.

Clearly, such classifications also require intimate knowledge of the language being examined. Importantly, however, as discussed in the section ‘Psycho- and neurolinguistic support’, the present framework is not intended to be specific to English or to languages that are structurally similar to English. On the contrary, the framework has already proven fruitful in predicting attentional patterns in structurally very different languages. Most recently, an eye-tracking study of reading in the polysynthetic language Kalaallisut (Greenlandic) found that readers allocated more attention to lexical than to grammatical suffixes within multi-morphemic words, when the suffixes were classified according to the theoretical criteria adopted here (Jaeger et al., sbm. ). This suggests that the distinction generalizes beyond the word level and beyond English. At the same time, the precise implementation of the classification necessarily depends on the structural properties of the individual language and requires language-specific analysis.

### Skipping and Attention

Though our reclassification of Staub’s target words was supported by the fixation time results, our hypotheses were not fully confirmed: no evidence was found for a difference in skipping probability (though there was a numerical trend in the expected direction of more skipping of grammatical words). Why might this be? Of course one factor is that our reanalysis was done on a smaller and less balanced subset of Staub’s data which makes results slightly less reliable. What we have presented above is merely a reanalysis of some of the data in Staub (2024). A better way to test this would of course be to design an entire new well-balanced study where categorization of words is based on the theoretically grounded criteria we have presented.

Another possibility is that the lack of effect on skipping has to do with how eye-tracking reflects attentional processes in reading. According to the usage-based theory outlined in Boye and Harder (2012); Boye (2023a, 2024), lexical elements attract more attention than grammatical elements in language perception. Attention can be understood as the allocation of cognitive resources to something (in line with Mole, 2021). This leads to the hypothesis that lexical elements will receive longer fixations than grammatical elements (as it is commonly agreed that fixation time reflects the amount of resources spent processing a stimulus).

Skipping, however, depends on parafoveal processing (Schotter et al., 2012), and most word skipping occurs when a word has been identified during fixation on the previous word (Gordon et al., 2013). The relationship between attention and skipping is therefore less direct than the relationship between attention and fixation times. While fixation time measures quite straightforwardly reflect processing effort, word skipping depends both on the degree to which a word has been parafoveally processed *and* on whether that processing is deemed sufficient for the word to be skipped. Based on the idea that grammatical words attract less attention, we would predict that grammatical words are skipped more often, because it may more often be “enough” for them to be only parafoveally processed and not directly fixated. However, this effect may be hard to detect due to multiple interacting factors, pertaining to both the target and previous word, which influence the extent of parafoveal processing. This makes skipping a noisier measure of attention allocation than fixation times.

The fact that we did not find a higher skipping probability for grammatical words also aligns quite well with Staub et al. (2019) who found that in sentences with a repeated *the* (e.g. *Amanda jumped off the the swing and landed on her feet.*), even when the word was fixated, participants did not necessarily detect the repeated word. This suggests that direct fixation does not necessarily imply attentive processing. In a similar study of Brazilian Portuguese, Vieira et al. (2025) found much higher detection rates for repeated articles than Staub et al. (2019) did for English, perhaps because Brazilian Portuguese articles encode more information than English articles, which would highlight the role of language-specific morphosyntactic properties in shaping attention to words. Notably, detection rates were high regardless of whether the repeated article was fixated or skipped during first-pass reading, further demonstrating that skipping behavior is not a straightforward indicator of attention allocation.

### Backgrounding of Lexical Words

It should be noted here that three of Staub’s content words (*girl*, *people*, *think*) show unusually short fixation times, even shorter than those of the grammatical words. We do not have any straightforward explanation of this, but we would like to stress that it is not incompatible with our main point. Our main empirical hypothesis is that grammatical words show shorter fixation times in general than lexical words, because grammatical words are conventionalized and hence entrenched as carriers of background information. Now, by the theory in Boye and Harder (2012), also lexical words have the potential to be backgrounded; the only difference from grammatical words is that they need not be. Thus, it is perfectly possible that lexical words are occasionally backgrounded to an extent that would result in short fixation times.

In fact, there are reasons to believe that this may be the case with *girl*, *people* and *think*. These words are frequently backgrounded in Staub’s stimulus material. For instance, three out of eight occurrences of *think* is in the clause *I think*, which is well known to function predominantly as a backgrounded, so-called “parenthetical” clause relative to an accompanying clause (e.g. Asher, 2000; Newmeyer, 2015; Boye & Harder, 2021). As the name suggests, parentheticals can be omitted without affecting the main points of the sentence, and this is exactly the case, for instance, with *I think* in Staub’s stimulus sentence in (8a), as illustrated in (8b).

- Either way I think he’s a prime candidate for a breakout season next year.

(8b) Either way he’s a prime candidate for a breakout season next year.

Similarly, several of the eight occurrences of each of the target words *girl* and *people* are clearly backgrounded. In (9a), *people* is backgrounded to the extent that it can straightforwardly be omitted altogether without making the sentence unacceptable or less informative, cf. (9b). The same goes for *a girl* in (10a), as shown in (10b). And in (11) *a girl* is not only repeated, but part of a tautological idiom the parts of which are arguably uninformative and backgrounded in themselves.

- It’s not easy for most people to know if their routers, DVRs, and other Internet-connected devices are infected.

(9b) It’s not easy for most to know if their routers, DVRs, and other Internet-connected devices are infected.

- Rogue wasn’t a shy and demure girl unsure of herself or her abilities.

(10b) Rogue wasn’t shy and demure, unsure of herself or her abilities.

(11) He leaves me no choice, and I’m sorry for it, but a girl must do what a girl must do.

This is however all speculations. Research investigating the effect on fixation times of different ways of foregrounding vs. backgrounding of lexical words is still sparse (e.g. Ganushchak et al., 2014)

## Conclusion

The main aims of the present paper were twofold: (1) to point to problems in the traditional distinction between content and function words, and (2) to argue that psycholinguistic studies must be based on theoretically coherent distinctions.

In pursuit of the first aim, we argued that the content-function word distinction is not only pre-theoretical and vague, but presupposes a distinction between ontologically different meaning types that is rejected by both proponents of functional linguistics (Harder, 1996) and leading figures in generative linguistics (Friederici et al., 2017). We also argued that the widespread understanding of the distinction as co-extensive with a distinction between open- and closed-class words is wrong, as demonstrated by converging results from works in, especially, aphasiology, linguistic typology and grammatical theory.

We discussed Staub (2024) as a case in point. Staub (2024) did not find evidence for any processing correlates of the distinction between content and function words. This might seem surprising, but it is really just another piece of evidence for the problematic status of the distinction, and from the point of view of a coherent and empirically supported theory of the lexical-grammatical contrast such as the theory proposed in Boye and Harder (2012), there is no surprise at all.

As for the second aim, our reanalysis of Staub’s data highlights the urgent need for psycholinguistics to move beyond the pre-theoretical distinction and to study the lexical-grammatical contrast on the basis of a coherent and empirically supported theory.

The fixation time results of our reanalysis support the claim that Staub’s (2024) function word group is heterogeneous, containing both words that are lexical and words that are grammatical by the theory in Boye and Harder (2012). The findings also suggest a difference in processing time between lexical and grammatical words that support the predictions entailed by that theory. As already emphasized, however, our reanalysis is based only on a subset of Staub’s materials. For this reason, it can and should be considered only as a supportive supplement to our main, conceptual aim which is to argue against traditional, theoretically unanchored distinctions between content and function words. Further eye-tracking research is needed to more precisely evaluate the effect of the lexical-grammatical contrast on reading measures.

## Data Availability

No new data were collected for this study. The analyses reported here are based on data originally collected by Staub (2024). The dataset and original analysis materials are publicly available from the repository linked in that article. The scripts used to produce the reanalyses reported in the present study are available at: https://github.com/solvej-jaeger/Reanalysis_Staub_2024.

## Footnotes

1 The logic here is that lexical elements attract more attention because they are entrenched with a greater attention-attracting potential as a consequence of their foregroundability.

2 All example sentences are experimental items from Staub’s Experiment 1.

3
https://dictionary.cambridge.org/grammar/british-grammar/some.
